# 4-Methyl-3-nitro­benzaldehyde

**DOI:** 10.1107/S1600536810033635

**Published:** 2010-08-28

**Authors:** Ze-Rong Guo, Hua-Bo Li, Fang Li

**Affiliations:** a State Key Laboratory of Explosion Science and Technology, Beijing Institute of Technology, Beijing 100081, People’s Republic of China

## Abstract

In the crystal structure of the title compound, C_8_H_7_NO_3_, mol­ecules are linked through weak inter­molecular C—H⋯O hydrogen bonding.

## Related literature

For the preparation, see: Johnson *et al.* (1991 ▶). For general background to supra­molecular electron-transfer materials, see: Yagi *et al.* (2003 ▶); Ezoe *et al.* (2006 ▶); Normand-Bayle *et al.* (2005 ▶); Ward *et al.* (2005 ▶). For a related structure, see: Zhang *et al.* (2009 ▶).
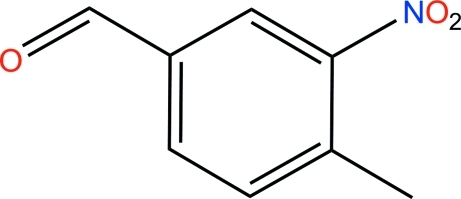


## Experimental

### 

#### Crystal data

C_8_H_7_NO_3_
*M* *_r_* = 165.15
Monoclinic, 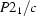

*a* = 3.9052 (6) Å
*b* = 17.841 (3) Å
*c* = 11.0663 (15) Å
β = 97.647 (2)°
*V* = 764.14 (19) Å^3^
*Z* = 4
Mo *K*α radiation
μ = 0.11 mm^−1^
*T* = 293 K
0.32 × 0.20 × 0.12 mm

#### Data collection

Bruker APEX CCD area-detector diffractometer
Absorption correction: multi-scan (*SADABS*; Bruker, 2003 ▶) *T*_min_ = 0.745, *T*_max_ = 1.000
4088 measured reflections
1353 independent reflections
1012 reflections with *I* > 2σ(*I*)
*R*_int_ = 0.018

#### Refinement

*R*[*F* ^2^ > 2σ(*F* ^2^)] = 0.039
*wR*(*F* ^2^) = 0.114
*S* = 1.05
1353 reflections
110 parameters
H-atom parameters constrained
Δρ_max_ = 0.13 e Å^−3^
Δρ_min_ = −0.16 e Å^−3^

### 

Data collection: *SMART* (Bruker, 2002 ▶); cell refinement: *SAINT-Plus* (Bruker, 2003 ▶); data reduction: *SAINT-Plus*; program(s) used to solve structure: *SHELXTL* (Sheldrick, 2008 ▶); program(s) used to refine structure: *SHELXTL*; molecular graphics: *ORTEP-3* (Farrugia, 1997 ▶); software used to prepare material for publication: *WinGX* (Farrugia, 1999 ▶).

## Supplementary Material

Crystal structure: contains datablocks I, global. DOI: 10.1107/S1600536810033635/lx2167sup1.cif

Structure factors: contains datablocks I. DOI: 10.1107/S1600536810033635/lx2167Isup2.hkl

Additional supplementary materials:  crystallographic information; 3D view; checkCIF report

## Figures and Tables

**Table 1:** Hydrogen-bond geometry (Å, °)

*D*—H⋯*A*	*D*—H	H⋯*A*	*D*⋯*A*	*D*—H⋯*A*
C4—H4⋯O3^i^	0.93	2.47	3.319 (2)	152

Symmetry code: (i) 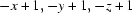.

**Fig. 1.**
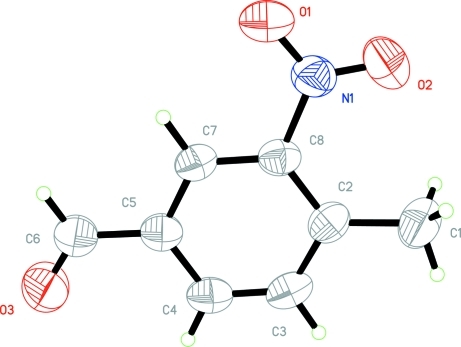
The molecular structure of the title compound with the atom numbering scheme. Displacement ellipsoids are drawn at the 50% probability level. H atoms are presented as a small spheres of arbitrary radius.

**Fig. 2.**
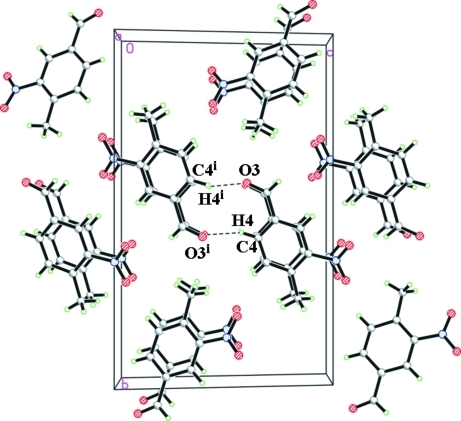
C—H···O interaction (dotted lines) in the crystal structure of the title compound.

## supplementary crystallographic information

### Comment

The title compound is an important intermediate for preparing supramolecular electron transfer materials (Yagi *et al.*, 2003; Ezoe *et al.*, 2006) and it has been utilized to synthesize medicinal compounds with biological activities. Herein we report the crystal structure of the title compound (Fig. 1).

The title compound crystallizes in the monoclinic space group *P2_1_/c*, the unit cell is consists of four molecules. In the title compound, the bond distances and bond angles are similar to those of the reported compound (Zhang *et al.*, 2009). The crystal packing (Fig. 2) is stabilized by a weak intermolecular C—H···O hydrogen bond between the benzene H atom and the oxygen of the aldehyde group(Table 1).

### Experimental

The title compound was obtained according to the literature method (Johnson *et al.*, 1991). Single crystals suitable for X–ray diffraction were prepared by slow evaporation of a solution of the title compound in diethyl ether at room temperature.

### Refinement

The H atoms were placed in calculated positions, with C—H = 0.93–0.96 Å and refined as riding modrl, with *U*_iso_(H) = 1.2–1.5 times *U*_eq_(C).

### Crystal data

**Table:**

C_8_H_7_NO_3_	*F*(000) = 344
*M**_r_* = 165.15	*D*_x_ = 1.435 Mg m^−^^3^
Monoclinic, *P*2_1_/*c*	Mo *K*α radiation, λ = 0.71073 Å
Hall symbol: -P 2ybc	Cell parameters from 1083 reflections
*a* = 3.9052 (6) Å	θ = 2.2–23.9°
*b* = 17.841 (3) Å	µ = 0.11 mm^−^^1^
*c* = 11.0663 (15) Å	*T* = 293 K
β = 97.647 (2)°	Block, colorless
*V* = 764.14 (19) Å^3^	0.32 × 0.20 × 0.12 mm
*Z* = 4	

### Data collection

**Table:**

Bruker APEX CCD area-detector diffractometer	1353 independent reflections
Radiation source: fine-focus sealed tube	1012 reflections with *I* > 2σ(*I*)
graphite	*R*_int_ = 0.018
φ and ω scans	θ_max_ = 25.0°, θ_min_ = 2.2°
Absorption correction: multi-scan (*SADABS*; Bruker, 2003)	*h* = −4→4
*T*_min_ = 0.745, *T*_max_ = 1.000	*k* = −21→18
4088 measured reflections	*l* = −13→13

### Refinement

**Table:**

Refinement on *F*^2^	Primary atom site location: structure-invariant direct methods
Least-squares matrix: full	Secondary atom site location: difference Fourier map
*R*[*F*^2^ > 2σ(*F*^2^)] = 0.039	Hydrogen site location: inferred from neighbouring sites
*wR*(*F*^2^) = 0.114	H-atom parameters constrained
*S* = 1.05	*w* = 1/[σ^2^(*F*_o_^2^) + (0.0555*P*)^2^ + 0.1376*P*] where *P* = (*F*_o_^2^ + 2*F*_c_^2^)/3
1353 reflections	(Δ/σ)_max_ < 0.001
110 parameters	Δρ_max_ = 0.13 e Å^−^^3^
0 restraints	Δρ_min_ = −0.16 e Å^−^^3^

### Special details

**Table:**

Geometry. All e.s.d.'s (except the e.s.d. in the dihedral angle between two l.s. planes) are estimated using the full covariance matrix. The cell e.s.d.'s are taken into account individually in the estimation of e.s.d.'s in distances, angles and torsion angles; correlations between e.s.d.'s in cell parameters are only used when they are defined by crystal symmetry. An approximate (isotropic) treatment of cell e.s.d.'s is used for estimating e.s.d.'s involving l.s. planes.
Refinement. Refinement of *F*^2^ against ALL reflections. The weighted *R*-factor *wR* and goodness of fit *S* are based on *F*^2^, conventional *R*-factors *R* are based on *F*, with *F* set to zero for negative *F*^2^. The threshold expression of *F*^2^ > σ(*F*^2^) is used only for calculating *R*-factors(gt) *etc*. and is not relevant to the choice of reflections for refinement. *R*-factors based on *F*^2^ are statistically about twice as large as those based on *F*, and *R*- factors based on ALL data will be even larger.

### Fractional atomic coordinates and isotropic or equivalent isotropic displacement parameters (Å^2^)

**Table:**

	*x*	*y*	*z*	*U*_iso_*/*U*_eq_	
O1	1.3758 (4)	0.60008 (9)	1.06104 (12)	0.0824 (5)	
O2	1.0886 (5)	0.70092 (9)	1.05565 (14)	0.0976 (6)	
O3	0.8183 (5)	0.42460 (9)	0.60139 (14)	0.0937 (6)	
N1	1.1695 (4)	0.64374 (9)	1.00814 (13)	0.0537 (4)	
C1	0.7588 (6)	0.75833 (11)	0.8424 (2)	0.0680 (6)	
H1A	0.6294	0.7831	0.7740	0.102*	
H1B	0.9756	0.7835	0.8638	0.102*	
H1C	0.6300	0.7594	0.9105	0.102*	
C2	0.8234 (4)	0.67836 (9)	0.80931 (16)	0.0479 (4)	
C3	0.6869 (5)	0.65315 (10)	0.69353 (16)	0.0549 (5)	
H3	0.5619	0.6866	0.6403	0.066*	
C4	0.7290 (5)	0.58141 (10)	0.65495 (15)	0.0546 (5)	
H4	0.6312	0.5669	0.5772	0.065*	
C5	0.9177 (4)	0.52988 (10)	0.73139 (14)	0.0483 (4)	
C6	0.9618 (5)	0.45197 (11)	0.69313 (17)	0.0640 (5)	
H6	1.1124	0.4217	0.7440	0.077*	
C7	1.0625 (4)	0.55297 (9)	0.84592 (14)	0.0458 (4)	
H7	1.1943	0.5197	0.8975	0.055*	
C8	1.0119 (4)	0.62533 (9)	0.88392 (14)	0.0440 (4)	

### Atomic displacement parameters (Å^2^)

**Table:**

	*U*^11^	*U*^22^	*U*^33^	*U*^12^	*U*^13^	*U*^23^
O1	0.0988 (12)	0.0828 (11)	0.0565 (9)	0.0162 (9)	−0.0233 (8)	−0.0012 (7)
O2	0.1323 (15)	0.0757 (11)	0.0757 (11)	0.0201 (10)	−0.0198 (10)	−0.0281 (9)
O3	0.1213 (14)	0.0757 (11)	0.0727 (10)	0.0141 (9)	−0.0293 (9)	−0.0225 (8)
N1	0.0589 (9)	0.0527 (9)	0.0476 (8)	−0.0074 (7)	0.0000 (7)	−0.0003 (7)
C1	0.0713 (13)	0.0501 (11)	0.0811 (14)	0.0055 (9)	0.0046 (11)	0.0069 (10)
C2	0.0438 (9)	0.0461 (10)	0.0540 (10)	−0.0021 (7)	0.0074 (8)	0.0087 (7)
C3	0.0530 (10)	0.0593 (12)	0.0504 (10)	0.0036 (8)	−0.0001 (8)	0.0172 (8)
C4	0.0554 (11)	0.0646 (12)	0.0412 (9)	−0.0009 (9)	−0.0028 (8)	0.0046 (8)
C5	0.0472 (10)	0.0536 (10)	0.0429 (9)	−0.0010 (7)	0.0011 (7)	0.0010 (7)
C6	0.0730 (13)	0.0625 (13)	0.0527 (11)	0.0082 (10)	−0.0063 (9)	−0.0047 (9)
C7	0.0439 (9)	0.0480 (10)	0.0438 (9)	0.0007 (7)	0.0000 (7)	0.0072 (7)
C8	0.0427 (9)	0.0488 (10)	0.0396 (9)	−0.0064 (7)	0.0028 (7)	0.0046 (7)

### Geometric parameters (Å, °)

**Table:**

O1—N1	1.2135 (19)	C3—C4	1.366 (3)
O2—N1	1.209 (2)	C3—H3	0.9300
O3—C6	1.197 (2)	C4—C5	1.392 (2)
N1—C8	1.467 (2)	C4—H4	0.9300
C1—C2	1.503 (2)	C5—C7	1.380 (2)
C1—H1A	0.9600	C5—C6	1.470 (3)
C1—H1B	0.9600	C6—H6	0.9300
C1—H1C	0.9600	C7—C8	1.380 (2)
C2—C3	1.395 (2)	C7—H7	0.9300
C2—C8	1.399 (2)		
			
O2—N1—O1	121.80 (16)	C3—C4—C5	120.32 (16)
O2—N1—C8	119.68 (16)	C3—C4—H4	119.8
O1—N1—C8	118.51 (15)	C5—C4—H4	119.8
C2—C1—H1A	109.5	C7—C5—C4	118.69 (17)
C2—C1—H1B	109.5	C7—C5—C6	119.82 (16)
H1A—C1—H1B	109.5	C4—C5—C6	121.49 (16)
C2—C1—H1C	109.5	O3—C6—C5	124.79 (18)
H1A—C1—H1C	109.5	O3—C6—H6	117.6
H1B—C1—H1C	109.5	C5—C6—H6	117.6
C3—C2—C8	115.51 (16)	C5—C7—C8	120.08 (15)
C3—C2—C1	118.29 (16)	C5—C7—H7	120.0
C8—C2—C1	126.21 (16)	C8—C7—H7	120.0
C4—C3—C2	122.81 (16)	C7—C8—C2	122.57 (15)
C4—C3—H3	118.6	C7—C8—N1	115.84 (14)
C2—C3—H3	118.6	C2—C8—N1	121.59 (15)
			
C8—C2—C3—C4	0.8 (3)	C5—C7—C8—N1	178.59 (14)
C1—C2—C3—C4	−179.54 (18)	C3—C2—C8—C7	0.4 (2)
C2—C3—C4—C5	−0.7 (3)	C1—C2—C8—C7	−179.27 (16)
C3—C4—C5—C7	−0.5 (3)	C3—C2—C8—N1	−179.79 (14)
C3—C4—C5—C6	178.88 (17)	C1—C2—C8—N1	0.5 (3)
C7—C5—C6—O3	172.0 (2)	O2—N1—C8—C7	−167.19 (17)
C4—C5—C6—O3	−7.4 (3)	O1—N1—C8—C7	11.8 (2)
C4—C5—C7—C8	1.6 (2)	O2—N1—C8—C2	13.0 (3)
C6—C5—C7—C8	−177.77 (16)	O1—N1—C8—C2	−167.98 (17)
C5—C7—C8—C2	−1.6 (3)		

### Hydrogen-bond geometry (Å, °)

**Table:**

*D*—H···*A*	*D*—H	H···*A*	*D*···*A*	*D*—H···*A*
C4—H4···O3^i^	0.93	2.47	3.319 (2)	152.

Symmetry codes: (i) −*x*+1, −*y*+1, −*z*+1.
